# Malnutrition in children under the age of 5 years in a primary health care setting

**DOI:** 10.4102/safp.v63i1.5337

**Published:** 2021-09-07

**Authors:** Indiran Govender, Selvandran Rangiah, Ramprakash Kaswa, Doudou Nzaumvila

**Affiliations:** 1 Department of Family Medicine, Sefako Makgatho Health Sciences University, Ga-Rankuwa, South Africa; 2 Department of Family Medicine, Faculty of Health Sciences, University of KwaZulu-Natal, Durban, South Africa; 3 Department of Family Medicine, Faculty of Health Sciences, Walter Sisulu University, Umtata, South Africa; 4 Department of Family Medicine, Faculty of Sciences, University of Pretoria, Pretoria, South Africa

**Keywords:** obesity, severe acute malnutrition, individual factors, community level factors, dietary intervention

## Abstract

In this study, we outlined the types of malnutrition amongst children, the causes of malnutrition intervention at the primary health care level and some recommendations to alleviate childhood malnutrition in South Africa.

## Background

Malnutrition is a health condition resulting from eating food that contains either insufficient or too many calories, carbohydrates, vitamins, proteins or minerals.^1,2^ It is a state of under- or overnutrition, evidenced by a deficiency or an excess of essential nutrients.^3^ Good nutrition is the basic need for children to thrive, grow, learn, play and participate. Section 28(1) (c) of the Bill of Rights in the South African Constitution guarantees every child the right to basic nutrition, shelter, basic healthcare services and social services that are related to the best interests of the child.^4^ Access of every child to sufficient food may be the responsibility of parents and child to determine the fulfilment of this right. Malnutrition often steals dreams from their young lives and hangs their future in the balance.^5^ It remains a major public health concern for children under the age of 5 years in many low- and middle-income countries because it is still the leading underlying cause of child mortality in these countries.^6^ Children are more vulnerable to macro- and micronutrient deficiencies caused by high demand for food during their years of growth.^6,7^ The effects of malnutrition in children under the age of 5 years include underweight, stunting, wasting with or without oedema (previously known as marasmus and kwashiorkor, respectively) and even death.^8^

**FIGURE 1:**
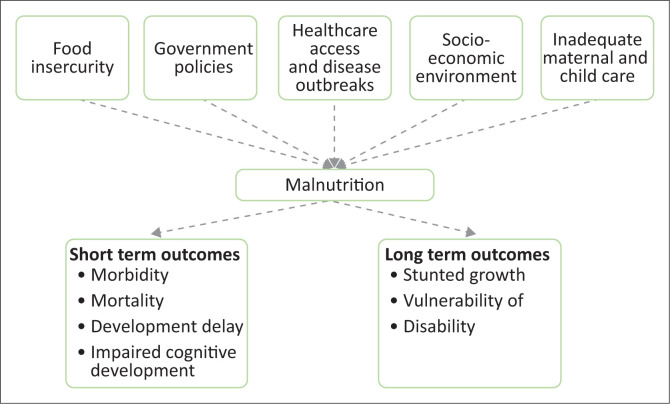
Outcomes of childhood malnutrition under the age of 5 years.

Malnutrition is the most severe consequence of food insecurity amongst children under the age of 5 years. Acute malnutrition can lead to morbidity, mortality and disability, as well as impaired cognitive and physical development with an increased risk of concurrent infections.^9^ Physical and mental health development is a fundamental right of a child, and their optimum level of health can be accessed with good nutritional support.^10^ Figure 1 demonstrates the consequences of malnutrition under the age of 5 years.

On 1 April 2016, the United Nations General assembly declared a decade of action on nutrition to address all forms of malnutrition by 2025.^11^ The Sustainable Development Goal (SDG)-2 (end hunger, achieve food security and improve nutrition), SDG-3 (ensure healthy lives and promote well-being for all ages) and the Global strategy for Women’s, Children’s and Adolescent’s health also set the relevant nutritional outcome targets by 2030.^12,13^

Despite the ample support from the United Nations International Children’s Emergency Fund (UNICEF), World Health Organization (WHO) and World Bank towards achieving nutritional freedom, we are still far from the world without malnutrition.^14^ The WHO report published in March 2020 revealed insufficient progress towards the World Health Assembly targets set for 2025 and the SDG set for 2030.^7^ According to the WHO 2020 report, about 144 million children under 5 years have stunted growth, 47 million children are wasted and 14.3 million are severely wasted, whilst 38.3 million are overweight or obese.^6^ According to the 2016 South Africa Demographic and Health Survey (SADHS), the prevalence rate of wasting was found to be 2.5% and underweight was 6%, whilst the stunting rate remained high at 27.0% amongst children under 5 years.^15^ Around 45% of deaths reported amongst children under the age of 5 years are linked to undernutrition.^6^

## Causes of malnutrition

**FIGURE 2:**
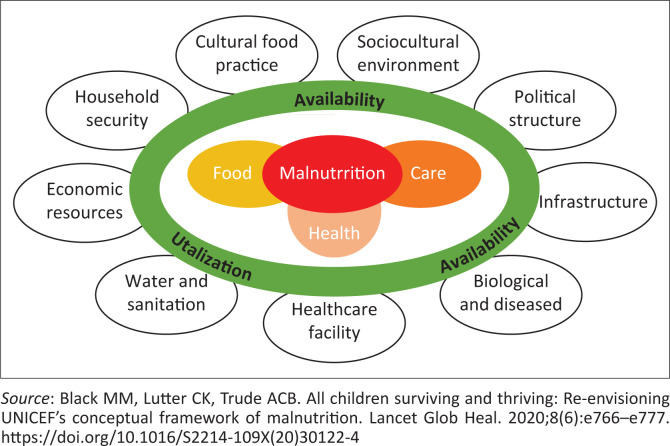
Theatrical framework for causes of malnutrition in children under the age of 5 years.

Malnutrition amongst children under the age of 5 years is a result of a complex interaction of availability, accessibility, and utilisation of food and healthcare services.^16^ Nutrition-specific factors include inadequate food intake, poor caregiving and parenting, improper food practices and infectious comorbidities. Nutrition-sensitive factors include food insecurity, inadequate economic resources at the individual, household, and community levels. Limited or poor access to education, healthcare services, infrastructure and poor hygienic environment are other nutritional sensitive factors that adversely affect the children under the age of 5-year nutritional status.^6,16^ Figure 2 demonstrates the theoretical framework for the causes of malnutrition under the age of 5 years. The major factors affecting the nutritional status of children under the age of 5 years are classified into the following three categories.

### Individual level factors

The risk factors for malnutrition on the basis of individuals include age, gender, birthweight, breastfeeding and childhood comorbidities. Teenage pregnancy, lower maternal education, low birthweight, lack of breastfeeding and personal food preference are also individual determinants of malnutrition of children under the age of 5 years.^17,18^ Although low birthweight is an individual factor, it is influenced by maternal health and nutritional status, as well as food security at the household or community level.

### Household-level factors

At the household level, age, gender, geographical area, level of maternal education, family income, household size, food security and healthcare access are important factors that had a significant association with child malnutrition.^18,19^ Malnutrition is an economic problem at the household level, which is accompanied by poverty, disturbed family structure, and ignorance of health and wellness of children. The National Income Dynamics Study – Coronavirus Rapid Mobile Survey (NIDS-CRAM) reported strong evidence of rapid increases in household and food insecurity during the coronavirus disease-19 pandemic.^20^ Lack of awareness of the nutritional quality of food, cultural and community beliefs about food and inappropriate feeding habits all lead to malnutrition amongst children under the age of 5 years.^17^ The nurturing care that children receive early in their life provides the basis for prospective nutritional status, with children of teenage mothers and younger household heads being more likely to be undernourished.^16^

### Community-level factors

The indicators of childhood malnutrition at the household level are influenced by place of residence, household infrastructure, income and ethnicity.^21,22^ The area of residence is a proxy indicator to determine the nutritional status of children for environmental risks, availability of health and wellness services, and shared community and cultural beliefs.^3^ Most of the South African villages have poor dwellings with poor access to basic services, including water, sanitation, electricity and healthcare facilities, which increased the risk of childhood malnutrition under the age of 5 years.^23^ The external force that influences food availability, accessibility and utilisation is highly influenced by politics, ideology, pandemics, economics and climate.^24^ Community wealth, community education level, prevalence of communicable diseases (e.g. human immunodeficiency virus [HIV], Tuberculosis [TB], etc.), and the distance of community to healthcare facilities also have a great influence on the child nutritional status.^17^

The theatrical framework for child malnutrition under the age of 5 years was adopted by UNICEF in 1990.^6^ It highlighted both basic and underlying causes of malnutrition, which includes the roles of inadequate dietary intake and healthcare received during childhood. The availability, accessibility and utilisation of food are highlighted as direct causes of malnutrition; however, the intermediate and underlying causes of malnutrition are multi-sectoral and extended to human, economic, household and community resources, influenced by geographical factors and economic structure.^25,26^ The adoption of the SDGs has brought global recognition of child nutrition, which was determined not only by children surviving but also by growth and thriving.^12^

## Patterns of malnutrition

**FIGURE 3:**
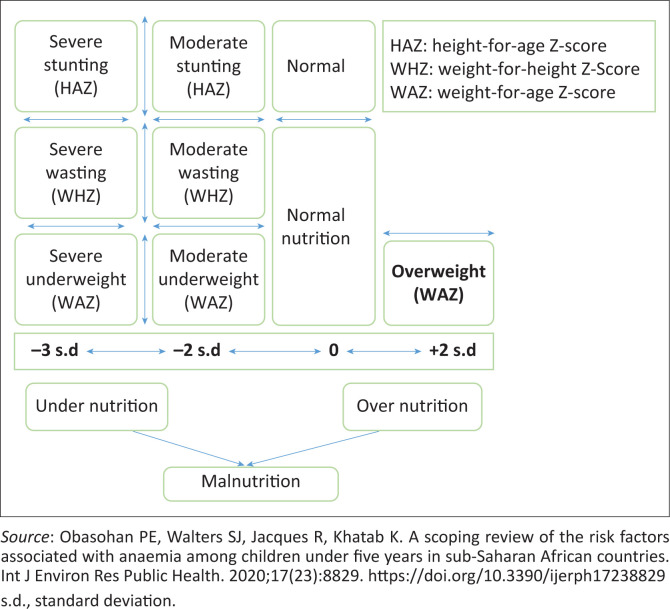
Pattern of malnutrition in children under the age of 5 years.

There is coexistence of substantial levels of undernutrition, particularly stunting and wasting, within the same geographical region, indicating the double burden of malnutrition.^27^ The patterns of nutritional status driven by nutrition transition, lifestyle changes, economic growth, social change and urbanisation occurred in South Africa.^15,23^ The nutritional status is also influenced by other factors at the individual, household and community levels. The WHO uses anthropometric indices to identify and categorise the nutritional status, which include height-for-age, weight-for-height and weight-for-age for measuring stunting, wasting and underweight. These indices are measured and compared as standard deviation units (Z-scores) from the median of the reference population.^6^ Figure 3 demonstrates the pattern of malnutrition in children under the age of 5 years.

## Stunting in children under the age of 5 years

Stunting (height-for-age) in a child is defined as too short for his or her age with a height-for-age Z-score less than −2 s.d. from the median of the reference population. It is an indicator of linear growth retardation and cumulative growth deficits in children because of chronic malnutrition.^5^ Stunting is usually associated with low socio-economic conditions, poor maternal health and nutritional status, inappropriate feeding and frequent hospital admissions in early life.^15^ Linear growth is a strong predictor of morbidity, mortality and learning capability during later life. Stunting is largely irreversible, especially the first 1000 days from conception have adverse effects in child’s life.^28^ It has a major influence on the physical and cognitive development of a child.^29^

According to UNICEF, WHO and World Bank Group 2020 report, an estimated 144 million children under the age of 5 suffer from stunting, globally. The stunting rates are decreasing in all regions worldwide, except for the African region that faces a rising number of stunted children.^14^ The number of stunted children under the age of 5 years in Africa has risen from 49.7 to 57.5 million between 2000 and 2019.^14^ During the same period, Southern Africa alone had reported the rise of 100 000 stunted under-5 years children.^6^

## Wasting in children under 5 years of age

Wasting in a child is defined as low weight-for-height, where the weight-for-height Z-score is less than −2 s.d. from the median of the reference population. Wasting demonstrates an acute undernutrition status that measures body mass with height and describes the current nutritional status of a child.^6^ It usually indicates recent and severe weight loss because of unavailability of enough food and infectious diseases, such as diarrhoea. A young child with moderate-to-severely wasted episodes has an increased risk of death.^3^

The main underlying causes of wasting include poor access to appropriate healthcare, lack of food security, inappropriate feeding practices, a monotonous diet with low nutrient density, and lack of water, sanitation and hygiene services. Severe wasting episodes weaken a child’s immunity, thereby making him or her susceptible to long-term developmental delays with an increased risk of death.^10^ According to the 2020 WHO report, of the 47.0 million children under the age of 5 years who were wasted, 14.3 million were severely wasted, with over a one-third of them living in Africa.^6^

### Underweight

Underweight amongst children under the age of 5 years is defined as low weight-for-age, with a Z-score of −2 s.d. from the median of the reference population. This condition is a composite extraction of both stunting and wasting, that is, an underweight child may be stunted, wasted or both.^30^

### Overweight

Overweight refers to a child whose weight-for-height Z-score is above two standard deviations (+2 s.d.) from the median of the reference population. Overweight is an emerging face of childhood malnutrition. There are reportedly now 38.3 million overweight children globally, an increase of 8 million since 2000. The rise of the overweight epidemic has been because of greater access to processed foods, along with lower levels of physical activity.^2^

### Severe Acute Malnutrition

Severe acute malnutrition (SAM) is a severe form of malnutrition defined as weight-for-height/weight-for-length, with a Z-score of −3 s.d. from the median of the reference population and the mid-upper-arm circumference of < 115 mm with bilateral nutritional oedema.^31^ Based on the current WHO guidelines, childhood malnutrition is broadly categorised into acute and chronic malnutrition. Acute malnutrition is further classified based on severity into moderate acute malnutrition (MAM) (weight-for-height/weight-for-length with Z-score between –3 s.d. and –2 s.d.) and SAM as defined above.^6^

### Interventions

Malnutrition is a complex issue that needs intervention beyond the healthcare facility, and a multisectoral holistic approach needed to be considered for the management of malnutrition in children under the age of 5 years.^32,33^ The primary health care worker is the first contact person for a health-related issue outside the household, and he or she plays a vital role in the management of malnutrition amongst children under the age of 5 years. With its historical background, characterised by a high level of inequality, high burden of TB and HIV/acquired immunodeficiency syndrome (AIDS) over the past few decades, rapid economic and social transition, and urbanisation in South Africa has created a complex health transition. It has resulted in high levels of persistent undernutrition amongst the lower income population potentially because of high levels of food insecurity at the household level.^10^ The following interventions might be considered to overcome the complex issue of malnutrition amongst children under the age of 5 years.^26,33^

## Community-based strategies

The community-based management of malnutrition enables community healthcare workers to identify and initiate treatment for children with malnutrition before they become seriously ill.^22^ This helps in the early detection of severe acute malnutrition in the community and the provision of management for those without medical complications.^19^ Ready-to-use therapeutic foods or other nutrient-dense foods are part of community-based strategies.^23^ Active community-based surveillance by community healthcare workers is the key to nutritional counselling, early identification and management of malnutrition.^30^ This approach provides an opportunity for a primary health care worker to understand the context of malnutrition that assists in the preparation of energy-dense child foods using locally available, culturally acceptable, and affordable food items.^23^ The community-based management of malnutrition can prevent both short-term and long-term consequences of childhood malnutrition.^10^

## Health facility-based strategies

The health facility-based strategy is being used in the management of acute malnutrition with medical complications. This approach can address therapeutic feeding, social assessment of the family to identify and address contributing factors.^19^ It also provides an opportunity to primary health care workers for counselling on appropriate feeding, care, and demonstration and practice of food hygiene.^30^ Early identification and prevention of low birthweight are part of basic antenatal care programmes in South Africa. Exclusive breast feeding, immunisation and complementary feeding are part of road-to-health card at the primary health care centre.^15^

## Nutrition-specific interventions

### Supplementary foods

Supplementary foods are ready-to-use, specially formulated, modified foods with energy density, protein, fat or micronutrient composition.^19^ They are designed to fulfil the nutritional requirements of specific populations.^31^ They are complementary foods intended for progressive adaptation of infants aged 6 months and older to family food. Supplementary food is used for the management of acute malnutrition with specific needs. Fortified blended foods and lipid-based nutrient supplements are examples of supplementary foods.^32^ In 1994, South Africa introduced a multi-sectorial Integrated Nutrition Programme (INP), which includes the Departments of Health, Social Development and Agriculture to address malnutrition.^34^

### Therapeutic foods

These foods are used in the treatment of severe acute malnutrition, which are specially designed for use in the stabilisation and rehabilitation phases in an inpatient setting, and ready-to-use therapeutic foods are used in the rehabilitation phase, usually in an outpatient setting.^19^ Feeding formulas, such as F-75 and F-100 therapeutic milk, are an example of therapeutic foods.^32^ In 2010, the nutritional therapeutic Programme (NTP) was launched to address malnutrition as a therapeutic measure.^34^

### Prevention

The manifestation of malnutrition can be multifaceted; however, the most frequent determinants of child malnutrition include poor dietary quality, suboptimal child-caring practices and repeated childhood infections.^2^ According to WHO child growth standards, all infants and children under the age of 5 years presenting to primary health care facilities should check for weight and length/height for age at each encounter to identify their nutritional status.^10,19^ The mid-upper arm circumference measurement can be used for screening and identifying children with SAM or MAM at healthcare facilities and community levels.^10^ Child immunisation against infectious diseases can prevent recurrent illness and improve nutritional status.^30^

Caregivers and family members of children under the age of 5 years presenting to primary health care facilities should receive counselling on the general nutritional demands of childhood, basic health and hygiene. Mother needs support for nutrition before and during pregnancy and lactation with exclusive breastfeeding in the first 6 months and continued breastfeeding until 24 months or beyond.^23^ A community-based malnutrition prevention approach includes access to basic health, water, hygiene, and sanitation services and opportunities for safe physical activity.^32^

## Recommendations

Primary health care settings are well positioned for identification and management of child malnutrition under the age of 5 years. The following recommendations are applicable for primary health care providers (Box 1), which are part of the South African INP, NTP and IMCI guidelines.^15,20,34^

BOX 1 Primary health care recommendations.

**Table:** 

1	Measurement of anthropometric data on each healthcare visit
2	Regular screening of nutritional status
3	Regular screening of acute and chronic childhood illnesses
4	Nutritional counselling of parents and caregivers
5	Access to water and sanitation
6	Good infant feeding practices with complimentary foods
7	Supplementary foods for moderate to severe malnourished children
8	Integrated management of childhood illness (IMCI)
9	Early identification and management of nutritional status according to the WHO guidelines

WHO, World Health Organization.

## Conclusion

Childhood nutrition is an integral component of a multifocal relationship with health, economic, social developments, and political system of the country. Child malnutrition under the age of 5 years has a great influence on the cultural, social, economic and community food practices. Unlike adults, the nutritional status of children is directly influenced by maternal health during pre-pregnancy, pregnancy and breastfeeding. Primary health care is the entry point for the fulfilment of community healthcare needs. Primary healthcare providers play a vital role in screening, early identification, appropriate referral and integrated management of malnutrition in children under the age of 5 years.
