# Erratum: Malnutrition in children under the age of 5 years in a primary health care setting

**DOI:** 10.4102/safp.v63i1.5416

**Published:** 2021-12-14

**Authors:** Indiran Govender, Selvandran Rangiah, Ramprakash Kaswa, Doudou Nzaumvila

**Affiliations:** 1 Department of Family Medicine, Sefako Makgatho Health Sciences University, Ga-Rankuwa, South Africa; 2 Department of Family Medicine, Faculty of Health Sciences, University of KwaZulu-Natal, Durban, South Africa; 3 Department of Family Medicine, Faculty of Health Sciences, Walter Sisulu University, Umtata, South Africa

In the published article, Govender I, Rangiah S, Kaswa R, Nzaumvila D. Malnutrition in children under the age of 5 years in a primary health care setting. S Afr Fam Pract. 2021;63(1), a5337. https://doi.org/10.4102/safp.v63i1.5337, there was an error in the affiliation for the last author. Instead of ‘Department of Family Medicine, Faculty of Sciences, University of Pretoria, Pretoria, South Africa’, it should be ‘Department of Family Medicine, Sefako Makgatho Health Sciences University, Ga-Rankuwa, South Africa’.

In addition, the incorrect ‘Authors’ contributions’ statement was given on page 5. The correct statement should read ‘All authors contributed equally to this manuscript’ instead of ‘I.O. and S.R. contributed equally to the design and implementation of the research, to the analysis of the results and to the writing of the manuscript’.

The publisher apologise for this error. The correction does not change the study’s findings of significance or overall interpretation of the study’s results or the scientific conclusions of the article in any way.

